# Strips of prairie vegetation placed within row crops can sustain native bee communities

**DOI:** 10.1371/journal.pone.0240354

**Published:** 2020-10-29

**Authors:** Farnaz Kordbacheh, Matt Liebman, Mary Harris

**Affiliations:** 1 Department of Agronomy, Iowa State University, Ames, Iowa, United States of America; 2 Department of Natural Resource Ecology and Management, Iowa State University, Ames, Iowa, United States of America; University of the Balearic Islands, SPAIN

## Abstract

As landscapes have become increasingly dominated by intensive agricultural production, plant diversity has declined steeply along with communities of pollinating insects including bees. Semi-natural habitats, such as field edge meadows and hedgerows, can be maintained to provide a diversity of flowering plants that can increase floral resources required by bees. An additional habitat enhancement practice is that of sowing strips of native prairie vegetation within row-cropped fields. In this study, conducted in Iowa, USA, we found that increases in both the abundance and diversity of floral resources in strips of native prairie vegetation within agricultural production fields greatly and positively influenced the bee community. The benefits to the bee community were important for both common and uncommon species and the effect may be strongest early in the season. Using networks of co-occurrence between plant and bee species, we were able to identify two native prairie plants, *Ratibida pinnata* and *Zizia aurea*, as potentially keystone resources that can be used to support native bees. When we evaluated the effect of reconstructed prairie strips on bees in the context of the surrounding landscape, we found that these conservation practices had positive effects on bees in agriculturally-dominated areas and that these effects were detectable in low to high complexity landscapes with 8–69% natural habitat. In landscapes dominated by crops with few pollen and nectar resources the inclusion of native prairie strips can buffer the decline of bees and effectively increase bee abundance and diversity.

## Introduction

Landscapes that have become dominated by crop production, particularly those crops with no dependence on animal pollination [1,2], have experienced declines in communities of native bees including unmanaged wild bees or non-*Apis* species [3], and other wild bee species important for pollination services [2,4–12]. Intensifying agricultural production affects some bee species more than others [13–15], and these species can differ greatly in the pollination services they provide [14]. Kleijn et al. [14] found that common bee species, those that constitute 2% or more of the relative abundance of individuals within communities, can provide as much as 80% of crop pollination services. These common species are important for pollination of crop species used for edible oils, protein, nuts, stimulants, fruits, and vegetables [6]. Common bee species from the families Halictidae, Apidae and Andrenidae that are found in crop fields can thrive despite habitat degradation [10,14,15], likely due to the more generalized utilization of floral resources [see 14]. Nonetheless, despite the resilience of common bee species against habitat degradation, these species have been reported to be vulnerable at a local scale in some areas [e.g.,^10^,^16^]. Uncommon bee species that are represented by few individuals and occur much less frequently in crop fields [14,15] are especially vulnerable to habitat degradation that occurs as the result of agricultural production [10,14,15]. The lack of robustness in uncommon bee species is possibly linked with their narrower diets and scarcity of specific floral resources throughout the landscape [5,10]. Uncommon bee species are important for stable pollination services to crops and for some endangered native plants [e.g., 8,10,17–20].

The utilization of native plants in flower-rich meadows, hedgerows, woodlands and other semi-natural habitats surrounding arable fields has been suggested as a way to mitigate the loss of bee species in agricultural landscapes. These practices enhance the landscape by providing pollen and nectar resources as well as nesting habitats for foraging pollinators [21–23], but their effects on bees in intensified agricultural landscapes can be species-specific [14,15]. In this regard, Kleijn et al. [14], and Kremen and M’Gonigle [15] showed that conservation of semi-natural habitat within intensified agricultural landscapes had stronger positive effects on uncommon bee species than common bee species. If conservation of natural habitat better supports uncommon bee species, refinements of this strategy that also support common bee species who actively provide the most pollination service would be desirable, especially in those spots of the landscapes that are at risk of losing these bee species.

The efficacy of habitat conservation practices aimed at improving pollinator communities should be studied within the context of the surrounding landscape [11,24]. Tscharntke et al. hypothesized that in situations where non-crop floral resources were severely limited or readily available (i.e., “cleared landscapes” with <1% non-crop areas or “complex landscapes” with >20% non-crop areas) habitat restoration would have few benefits to pollinator communities. However, within “simple landscapes” with 1–20% non-crop areas, they suggested that habitat management would have maximum benefits for the pollinator community [15,24]. The authors also stated that this relationship may vary in different regions of the world. Consequently, this hypothesis needs to be tested in a range of landscapes and geographical settings, including within areas of intensified agricultural production with crops that are non-dependent on pollinators [2].

In the midwestern U.S. state of Iowa, two crops, corn (*Zea mays* L.) and soybean (*Glycine max* (L.) Merr.), together cover more than 75% of the landscape (Cropland Data Layer) [25]. Neither of these crops requires animal-mediated pollination and they provide little pollen and nectar for bees [2,26]. Historically, Iowa was predominantly covered by tallgrass prairie vegetation composed of native perennial grass and forb species, many of which have been shown to provide high quality habitat and floral resources with which to support species-rich bee communities [27–29]. Over the last century, the conversion of a high proportion of prairie land in Iowa and other Midwestern states to corn and soybean production [30–32] has greatly reduced the habitat and floral resources important for the support of bees [1,2,28]. Zhou et al. [33] and Schulte et al. [34] described the use of reconstructed prairie communities in Iowa to improve soil, water, and biotic conservation, including increases in plant and bee abundance. This practice involves conversion of ~10–12% of the area within a row-crop production field to strips of native prairie vegetation. How this land use change can influence the structure of bee communities in terms of common and uncommon species is not yet well understood.

Critical habitat components that determine bee community structure are the quality and quantity of floral resources (i.e., nectar and pollen) [4,35], and temporal patterns of blooming throughout the season [36,37]. In this regard, bee-plant interaction networks can be valuable for assessing habitat restoration practices [38,39]. Methods relevant for elucidating these interactions include collecting flower visitors from individual plants [40], examining pollen loads of bee specimens [41], and direct field observation of flower visitations. These methods are labor- and time-intensive, and they can be skewed toward the most frequent interaction [42]. Direct observation can lead to over-collecting bees from highly abundant plant species and under-collecting bees from rare plant species. Thus, the bee-plant networks constructed from field observations may not estimate the true complexity of the community [42].

In this study, we examined the benefit of native prairie vegetation sown in corn and soybean fields for common and uncommon bee species at four sites within the state of Iowa. We predicted that adding strips of native prairie vegetation to corn and soybean fields in intensified agricultural landscapes would (i) increase overall bee community abundance, richness and diversity, and (ii) increase the abundance and richness of both common and uncommon bee species. To examine the effect of particular plant families or species for specific members of the bee community, we developed co-occurrence networks [43], using bee and plant species data. We expected that (iii) particular components of the plant community would have stronger associations with less common members of the bee community.

## Materials and methods

### Study sites and experimental design

Our study was conducted in 2016 and 2017 at four paired comparison sites (i.e., 8 crop fields) in Iowa located in Guthrie (site 1), Linn (2), Marshall (3), and Pottawattamie (4) counties. Permission to work at sites 3 and 4 was provided by Iowa State University; permission to work at sites 1 and 2 was provided by the private landowners. Our field work did not involve endangered or protected species. Sites were situated in landscapes dominated by intensively managed agricultural fields producing corn and soybean. At each paired study site, bees were sampled in two crop fields. The first field was 100% crop production and provided the control treatment. The second field also was used for crop production, however, 10–12% of the field area had been seeded with a diverse mix of native perennial prairie species within strips planted on the contour; this type of field represented the strip treatment.

For treatment comparisons, we did not sample bees in the cropped portion of fields with prairie strips due to their close proximity and the possibility of bees present in strips being attracted to traps in crops. Instead, two fields separated by 1 to 3 km were utilized, which is beyond the flight range of the majority of bee species, with “only few individuals such as bumble bees having the capability to cover long foraging distances of 1.5 km” [44–47]. This separation was intended to enhance the independence of bee communities in the control and strip treatments while allowing each field to be subject to the same environmental conditions.

Crop identity was the same at each site for strip and control fields, but the crop differed between locations; site 1 produced corn in 2016 and soybean in 2017, sites 2 and 4 produced soybean in 2016 and corn in 2017, and site 3 produced corn in both years. Prairie vegetation within strips at each site was in the early stages of establishment, ranging from one to three years post-seeding.

### Bee and plant community assessments

Bees at all sites were sampled four times each year, with approximately one month between samples, from late May until late August. Each sample was conducted on sunny days when temperatures were above 12.8°C and wind was below 24 km/hr to ensure that conditions were appropriate for bee flight. We sampled the two fields of a paired comparison site on the same day at the same time or within a window of two hours. For the latter case, we alternated sampling fields in a randomized order at different periods of sampling, so that bees experienced approximately similar temperatures at the time of sampling. Bees were captured along 60-m transects established at the field edge and in the middle of prairie strips in fields used for the strip treatment, and along the edge of control crop fields and within 50 m distance from the edge of the fields for the control treatment. We used three complementary collection methods–blue vane trapping, pan trapping, and aerial sweep netting–to more completely sample the bee community [37].

Blue vane traps were located at the beginning and end of the 60-m transects. The transects contained 12 pan traps (30 ml, SOLO^®^ brand, white plastic soufflé cups, Food Service Direct, Hampton, VA, USA), four each of which were painted fluorescent yellow and fluorescent blue (East Coast Guerrra Paint and Pigment, NY, USA) or left white. These traps were placed every five meters in random color order, partially filled with a weak detergent solution and left in place for six hours until collection [48–50]. Aerial sweep netting was conducted for 24 minutes with two sweepers walking the transect, sweeping through the vegetation. All trapped or netted bees, including *Apis mellifera* (Linnaeus) and wild unmanaged bees, were identified to species [51–53] except those in the *Lasioglossum* subgenus *Dialictus*. In this taxon, there are 97 species found east of the Mississippi River [54]. Gibbs described their identification as “taxonomically challenging” and they are often considered together as a single taxon. The identified bee species were partitioned into common and uncommon species using a ~1% abundance boundary, with *S* as the total species richness [55].

We evaluated floral cover of forbs, but not grasses, within the prairie strips of each study site, as grasses have been shown to provide no resources for bees [56]. At each study site, on the day bees were sampled, we estimated the percent cover of each blooming forb species within 10 1-m^2^-quadrats placed at 5-m intervals along the 60-m transect. We also measured the diversity of vegetation in the surrounding landscape. We calculated landscape diversity using the Shannon diversity index (H’s = -∑((p_i_) × ln (p_i_))), where p_i_ is the proportional abundance of i^th^ vegetation category among land cover categories (see S5 Table), within a 3-km radius of each treatment at each site with data obtained from the USDA National Agricultural Statistics Service Cropland Data Layer (https://nassgeodata.gmu.edu/CropScape/) [57]. To test whether there was a difference between the landscape diversity surrounding each treatment at a site, we performed a two-tailed t-test [58].

### Data analyses

#### Assessing the effect of prairie strips on bee community abundance and richness

We compared the response variables of bee abundance and species richness (by common species, uncommon species or families) between treatments, using generalized linear mixed models (GLIMMIX procedure, [59]). We first constructed our model with Poisson, negative binomial, and gamma distributions, all of which resulted in over-dispersion. We then used a normal distribution in which the response variable was transformed (ln (x+0.5)). Using this model, a good fit was obtained, and the assumptions of normality of residuals and homoscedasticity of variance were met. In this model, fixed effects were site, treatment, sampling month, and year, with random effects of site nested within year and treatment and site nested within year.

We tested whether the abundance and species richness of bee families differed across sampling months using the same mixed-model approach. When the effect was significant, we performed a post-hoc Tukey test for multiple comparisons among means (GLIMMIX procedure, [59]).

#### Bee community composition and evenness between treatments

The effect of treatment on the bee community was evaluated using two approaches. First, dissimilarities between the composition of communities were tested using the multi-response permutation procedure (MRPP) with Bray-Curtis as the distance measure (PC-ORD v.7) [60]. In the second approach, beta dispersion (species evenness, hereafter) was tested by multivariate dispersion analysis (MDR) in which the evenness of individuals among sampling units was compared while controlling for changes related to each species’ proportional abundance, as well as changes in overall species richness [61]. For this, Bray-Curtis distances between sample units within each treatment were calculated using the vegdist function of Vegan package in R v.3.3.1, and principal coordinates analysis (PCoA) was used to calculate the mean distance of each species to each treatment centroid in multidimensional space using betadisper function of Vegan package in R [62,63]. The mean distances of species from treatment centroids were subjected to analysis of variance to identify significant differences [63]. We categorized bee communities using a 1% boundary, with *S* as the total species richness found over the entire study [56]. Species that individually constituted >1% of relative abundance of the entire community were considered as common. Species that individually accounted for <1% of the bee community were delineated as uncommon. The *Lasioglossum* (Dialictus) species assemblage members were ubiquitous among our experimental sites. Therefore, we used two approaches with regard to the analysis of common species: (i) we removed the *Lasioglossum* (Dialictus) group from the larger common species group to avoid possible masking effects of these species on the other most common species; and (ii) we included *Lasioglossum* (Dialictus) group in the class of common species.

To determine the importance of common species, uncommon species, and all species together to overall community diversity we calculated sample size rarefaction curves, using three diversity measures, according to Hill’s series (ggiNext package in R) [63–66]. It has been suggested that if uncommon species are expected to be more important in determining community species richness, S, the Hill number 0, should be selected [66]. If uncommon and common species are expected to be equally important in driving changes in the Shannon diversity of the community, H’, the Hill number 1, should be used. If only common species are expected to drive changes in the Simpson’s diversity of the community, D, the Hill number 2 should be utilized [66]. We used all three Hill numbers to gain insight into the contribution of common and/or uncommon species.

#### Effect of treatment, landscape diversity and prairie vegetation on the bee community

We used linear regression models to examine the main and interactive effects of landscape diversity and prairie strip treatment on bee abundance and species richness of all bees, common bees and uncommon bees. Our model included terms for Shannon landscape diversity within a 3-km radius from each field, prairie strip treatment, and the interaction of landscape and strip treatment [58].

#### Plant and bee community correlations

The interaction between bee and plant species is usually measured by networks of observed bee-plant visitation. Here we developed a correlation-based network of co-occurrence from pair-wise correlations between bees and plants from monthly surveys in strip fields. One side of the correlation was abundance of each bee species per month per site; the other side of the correlation was proportional cover of each plant species per month per site. This approach has been used previously to study other organisms, such as microbes, that are difficult to observe directly [43]. To assess the potential importance of each blooming forb species within the strips for each identified bee species, we estimated pairwise correlations, setting r Pearson > 0 and *p* < 0.05 as the selection criteria to determine the significant positive correlations [58].

After obtaining pairwise correlations, we obtained a full matrix containing the number of pairwise correlations from possible bee-plant interactions. On this matrix we performed a Benjamini and Hochberg (BH) False Discovery Rate correction (FDR) [43,67] to adjust the *p* values due to the numerous correlations. FDR increased the *p* values, and consequently many of correlations between abundant bee and plant families that were observed in the field became statistically insignificant. To indicate if the primary bee-plant correlation matrix was robust to be used without adjustment using the FDR test, we tested the match between the primary bee-plant correlation-based network and the observed bee-plant visitation network. The observed bee-plant visitation data were collected by capturing bees visiting plants at identical times and locations as for data concerning bees captured in traps and available plant species. This matching was conducted by a separate Pearson correlation test between the entire matrix of bee-plant correlation-based data (co-occurrence network) and the entire matrix of observed bee-plant visitation data (interaction network). We found that our correlation-based network strongly matched the observed visitations. Therefore, we used the unadjusted co-occurrence network in this study to determine floral fidelity from May-August of 2016–2017 at four study sites.

To determine the association of bee groups with native prairie or exotic and weedy species, we grouped the correlations. The plant side of the correlations was classified as native prairie and exotic/weedy species and plant families; the bee side of the correlations was categorized by abundance (common and uncommon) and family (Apidae, Halictidae, Andrenidae, Colletidae and Megachilidae). We calculated the ratio of correlations between each bee group with native prairie plants to that of exotic/weedy plants.

To elucidate the importance throughout the season of plant families for bee families we used the significant positive correlations for each month to construct bipartite networks of co-occurrence (plotweb function of the bipartite package in R; [63–68]). In the generated networks, the length of the plant rectangle represents the relative frequency of significant correlations among all bee species, while the length of the bee family rectangle represents the relative frequency of significant correlations among all plant species. Link widths represent the magnitude of co-occurrence of a bee and a plant family, with wider links representing frequent co-occurrence and narrower links less frequent co-occurrence.

## Results

### Bee and plant communities

A total of 5,088 individual bees was collected over the entire study. We identified 2,969 individuals across 88 species and placed the remaining specimens into a single subgenus, *Lasiglossum (Dialictus*) (S1 Table). There were 8 species, all members of the Halictidae or Apidae families, that individually constituted >1% of relative abundance of the entire community and were thus delineated as ‘8 common bees’. Individuals placed in the subgenus *Lasioglossum* (Dialictus) very likely represented many unidentified species, but were ubiquitous among our experimental sites, and were treated as a single taxon. This taxon also constituted >1% of relative abundance of the entire community, and therefore we considered them as common species and added a second class of common species that included *Lasioglossum* (Dialictus), which will be referred to as ‘9 common bees’ hereafter. The remaining 80 species that individually accounted for <1% of the bee community were delineated as uncommon bees. All bees collected were members of the following families in order of relative abundance: Halictidae, Apidae, Andrenidae, Colletidae and Megachilidae.

The strips contained 55 blooming forb species (S2 Table), among the following families in order of relative cover/1 m^2^: Asteraceae, Fabaceae, Amaranthaceae, Lamiaceae, Polygonaceae, Apiaceae, Plantaginaceae, Onagraceae, Brassicaceae, Malvaceae, Apocynaceae, Rosaceae, Urticaceae, Scrophulariaceae, Oxalidaceae, Ranunculaceae, and Euphorbiaceae. There were relatively equal numbers of prairie species (27) and exotic/weedy species (28). Shannon landscape diversity estimates were relatively low and did not differ among sites nor between treatments except at the Linn County site where the landscape diversity surrounding the control treatment was greater than that of the strip treatment (1.153 and 1.005, respectively; *t-test*: *t*_1_ = 25.86, *p* = 0.0205; see S3 Table).

### Assessing the effects of prairie strips on bee community abundance and richness

Comparison of the bee community within prairie strips to that within controls showed that the strip treatment significantly increased bee abundance and species richness. We observed 1.6 times more individual bees (abundance) in the strips relative to the controls (3,199 vs. 1,887; *F*_1,6_ = 10.96, *p* = 0.016). The strip treatment also supported 1.7 times more bee species (81 vs. 46) (*F*_1,6_ = 19.62, *p* = 0.004). Furthermore, the strip treatment significantly enhanced the abundance and species richness of bees within all families (Table 1). The Apidae and Halictidae contained both common and uncommon species, whereas the other bee families, Andrenidae, Colletidae, and Megachilidae, consisted entirely of uncommon species.

**Table 1:** Bee abundance and species richness of each family in strip and control treatments across four sites during May-August of 2016 and 2017 from all trapping methods.

Family	Abundance					Species richness				
	Mean					Mean				
	strip	control	SE	*F*_1,6_	*p* value	strip	control	SE	*F*_1,6_	*p* value
Apidae	1.83 (5.71)	1.33 (3.26)	0.18	11.89	0.014	1.2 (2.82)	0.61 (1.34)	0.103	36.58	0.001
Halictidae	4.02 (55.36)	3.15 (22.91)	0.25	9.46	0.022	1.84 (5.83)	1.49 (3.95)	0.107	12.32	0.013
Andrenidae	0.43 (1.03)	-0.57 (0.06)	0.25	7.79	0.032	0.13 (0.64)	-0.59 (0.05)	0.179	7.41	0.035
Colletidae	-0.11 (0.40)	-0.64 (0.03)	0.12	6.21	0.047	-0.27 (0.26)	-0.66 (0.02)	0.09	7.14	0.037
Megachilidae	0.02 (0.52)	-0.57 (0.06)	0.1	31.51	0.001	-0.03 (0.465)	-0.59 (0.05)	0.089	34.87	0.001

Mean estimated values from generalized linear model analysis of variance. Ln (x+0.5) transformed and back-transformed means (in parentheses) are provided. SEs of mean differences *F*_df_ statistic and *p* values for paired comparisons are also presented.

**Fig 1:**
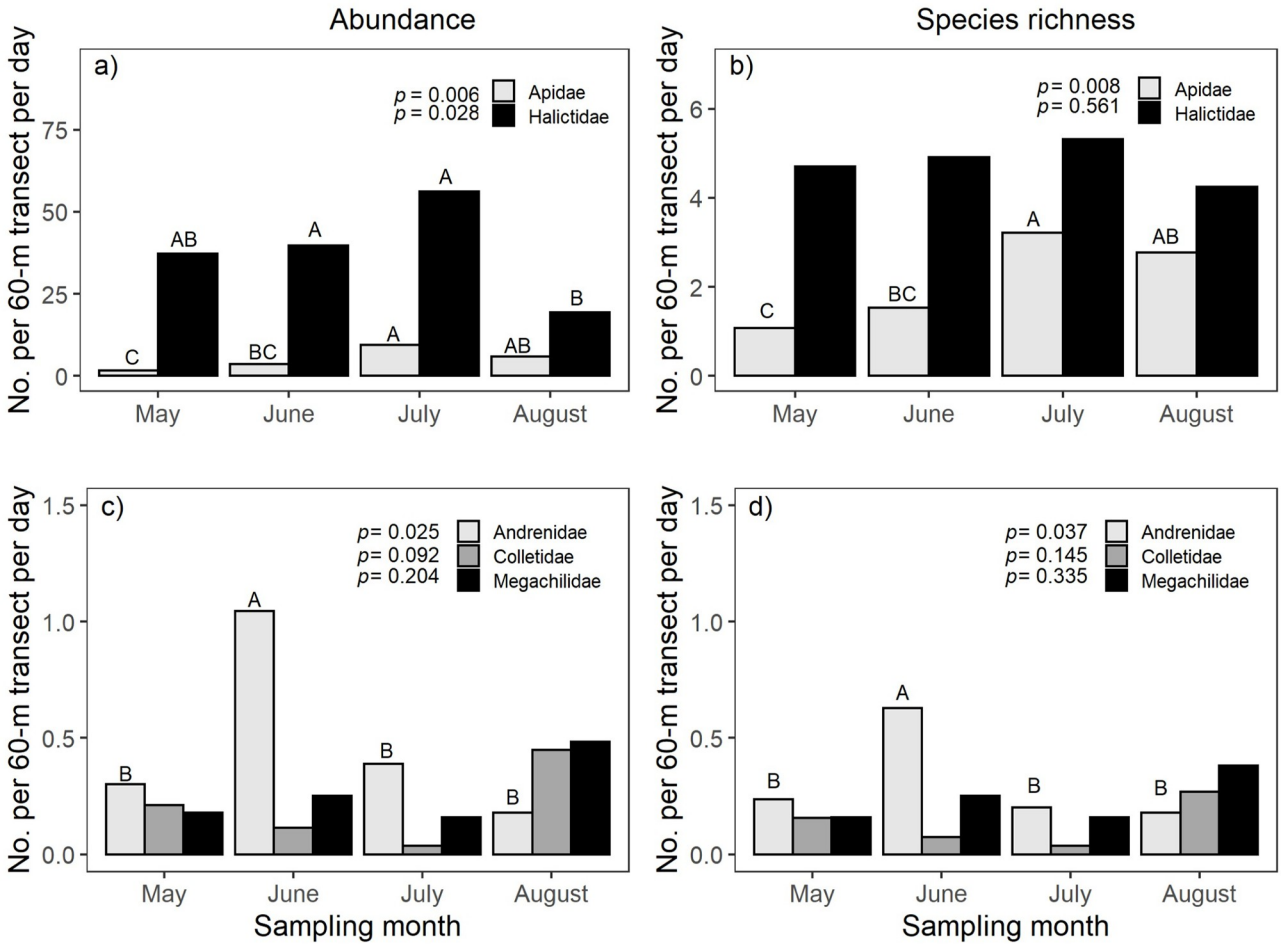
Monthly changes in abundance (a) and species richness (b) of bees in Apidae and Halictidae, and abundance (c) and species richness (d) of bees in Colletidae, Megachilidae, and Andrenidae. For each family, different letters above month bars indicate significant differences (*p* < 0.05). Values presented are back transformed from natural log values.

Our findings also indicate that the abundance and richness of some bee families, but not all, differed among months. The overall abundance of members of both the Apidae and Halictidae was highly affected by sampling month (*F*_3,36_ = 4.90, *p* = 0.006 and *F*_3,36_ = 3.41, *p* = 0.028, respectively), with the highest activity of each family occurring in July (Fig 1a; S4 Table). Similarly, species richness in the Apidae was greatest in July and August (*F*_3,36_ = 4.51, *p* = 0.008). However, species richness of the Halictidae did not differ across months as this family included the taxon of *Lasioglossum* (Dialictus) spp., for which we pooled the abundance of all individuals and counted them as one species. The presence of *Lasioglossum* (Dialictus) spp. in Halictidae, during the course of the experiment may have resulted in an even distribution of species richness within this family throughout the season (Fig 1b; S4 Table). Among families represented exclusively by uncommon species, the Andrenidae was most abundant and species rich in June (*F*_3,36_ = 3.51, *p* = 0.025 and *F*_3,36_ = 3.13, *p* = 0.037, respectively) (Fig 1c and 1d; S4 Table). The other families of uncommon species, the Colletidae and Megachilidae, were similarly abundant and species rich throughout the season, however, abundance and species richness were strikingly lower than those of the families of common bees.

### Bee community composition and evenness between treatments

Bee community composition was greatly affected by prairie strip treatment (MRPP; *T* = -2.23, *A* = 0.016, *p* = 0.039). The abundance and species richness of uncommon bees were significantly higher for the strip treatment than the control (*F*_1,6_ = 7.91, *p* = 0.031 and *F*_1,6_ = 12.42, *p* = 0.012, respectively). For the 8 common class analyzed as a group, the abundance and richness were significantly greater in the strip treatment than the control (*F*_1,6_ = 12.69, *p* = 0.012 and *F*_1,6_ = 7.09, *p* = 0.037, respectively). The inclusion of taxon *Lasioglossum* (Dialictus) spp. in the class of common species, presented as 9 common bees, again resulted in a significantly greater abundance and richness in the strip treatment than control (*F*_1,6_ = 7.01, *p* = 0.038 and *F*_1,6_ = 6.11, *p* = 0.048, respectively).

**Fig 2:**
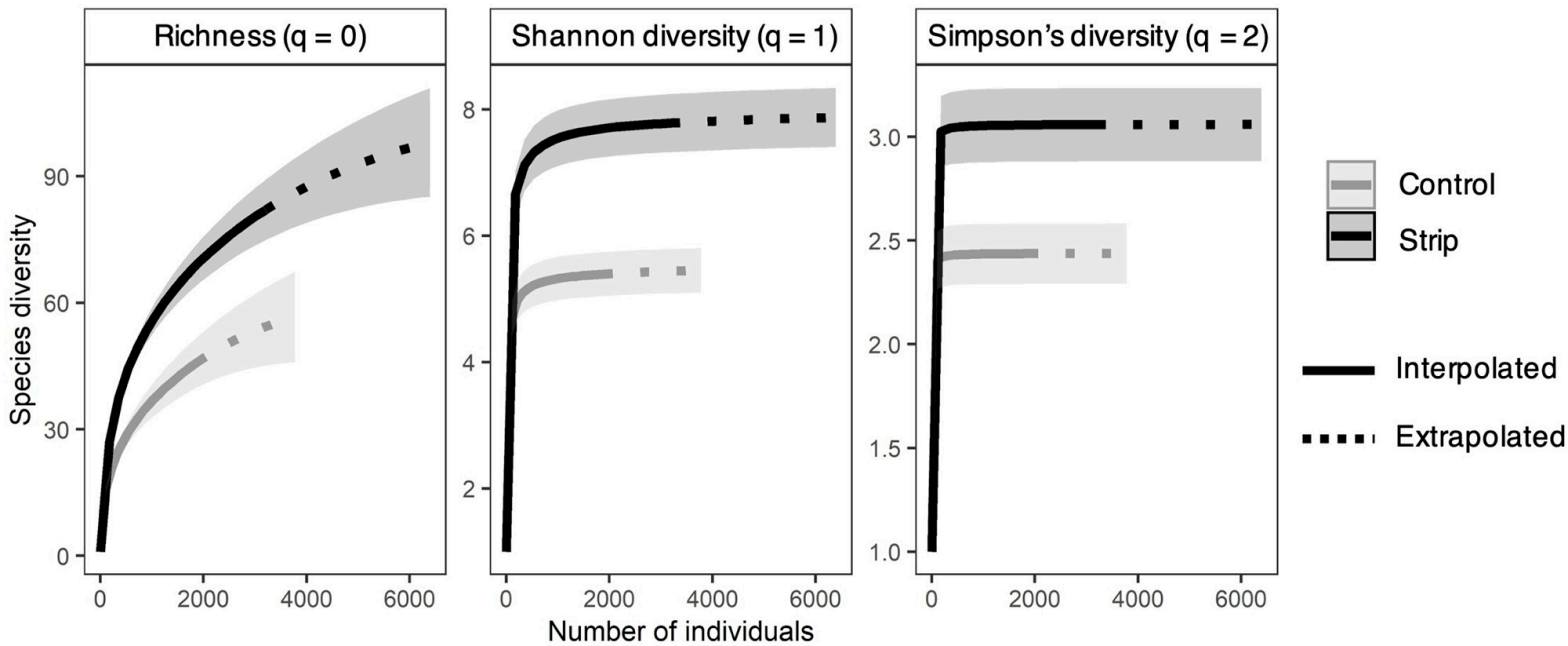
Sample-size-based rarefaction (solid lines) and extrapolation (dotted lines) curves with 95% confidence intervals (shaded areas) for the strip and control treatments by diversity order: a) q = 0 (species richness), b) q = 1 (Shannon diversity) and c) q = 2 (Simpson’s diversity).

There was no significant difference in bee community evenness between strip and control treatments (*F*_1,62_ = 2.45, *p* = 0.123) when the subgenus *Lasioglossum* (Dialictus) spp. was included in the analysis, possibly due to the masking effect of individuals placed in this group on other species. However, by excluding the subgenus *Lasioglossum* (Dialictus) spp. from the analysis, we detected a significant difference in bee community evenness between the strip and control treatments (*F*_1,62_ = 6.16, *p* = 0.016), with a higher evenness for strip than control. The latter result indicated a balance in distribution of abundances among bee species of the strip compared to the control treatment. Use of rarefaction curves for different diversity indices by Hill numbers enabled us to determine that both common and uncommon species contributed to higher bee community diversity in the strip treatment relative to the control (Fig 2). The species richness curve (q = 0) clearly indicated that additional sampling in the strip treatment could quickly capture a higher number of individuals of uncommon bee species and that the likelihood of collecting additional uncommon species was greater among strips than controls (Fig 2a). The strip treatment also resulted in higher bee diversity than that of the control as shown by the greater Shannon (q = 1) and Simpson’s (q = 2) diversities, indicating that strips had greater numbers of uncommon as well as common species (Fig 2b and 2c).

### Effect of treatment, landscape diversity, and prairie vegetation on the bee community

**Fig 3:**
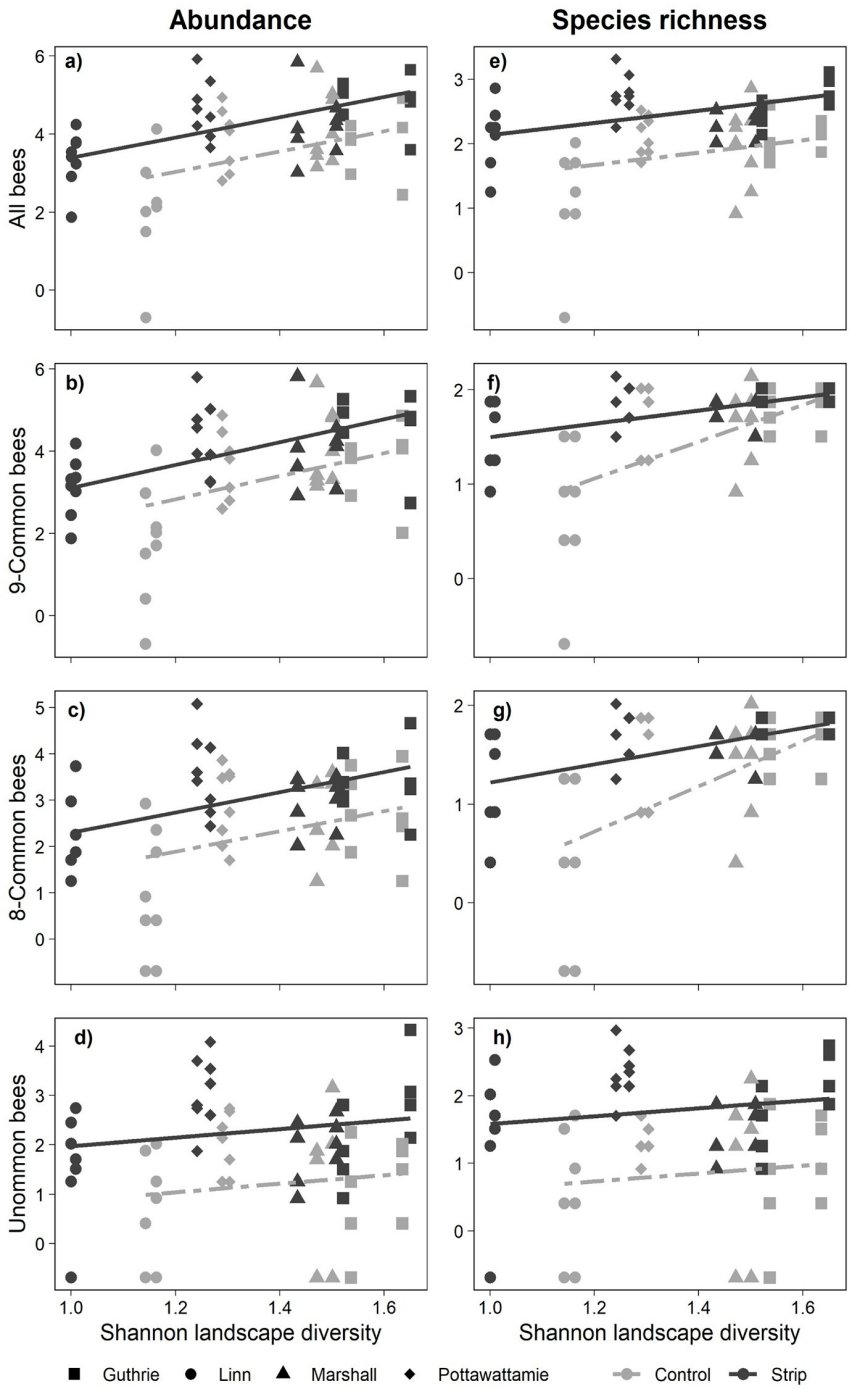
Bee abundance and species richness as affected by treatment (+/- prairie strips), Shannon landscape diversity and the interaction of landscape diversity with treatment for all bees (a and e), 8 common bees (b and f), 9 common bees, which represents 8 common species plus all individuals in subgenus *Lasioglossum* (Dialictus) spp. (c and g), and uncommon bees (d and g). Both abundance and species richness data, presented as ln(x+0,5)-transformed, are based on the number of bees per 60-m transect per day. Solid and dashed lines represent the strip treatment and control respectively. See S6 Table for results of statistical analyses.

Regression analyses with the presence or absence of prairie strips and Shannon landscape diversity as independent variables indicated that strip treatment had a highly significant effect on the abundance of all bees (Fig 3a and S6 Table; *p* = 0.0006), 8 common bees (Fig 3b; *p* = 0.0014), 9 common bees (Fig 3c; *p* = 0.0022), and uncommon bees (Fig 3d; *p* = 0.0002). Similarly, strip treatment significantly affected species richness among all bees (Fig 3e; *p* < 0.0001), 8 common bees (Fig 3f; *p* = 0.0003), 9 common bees (Fig 3g; *p* = 0.0004), and uncommon bees (Fig 3h; *p* < 0.0001). These results agree with those from the previous mixed model analyses and species composition analysis. Landscape diversity also significantly affected both abundance and species richness among all bees (*p* < 0.0001 to 0.0017), 8 common bees (0.0005 to < 0.0001) and 9 common bees (< 0.0001 to < 0.0001). The magnitude of the difference between strips and non-strip treatments varied only for the richness of common bee species (both 8 common and 9 common) across landscape gradients (Fig 3f and 3g; *p* = 0.0373 and 0.0234): here both strip and control reached the same level of richness where the landscape diversity was the highest. However, neither the abundance nor species richness of uncommon bees were affected by landscape diversity (Fig 3d and 3h; *p* = 0.278 and 0.265, respectively).

**Fig 4:**
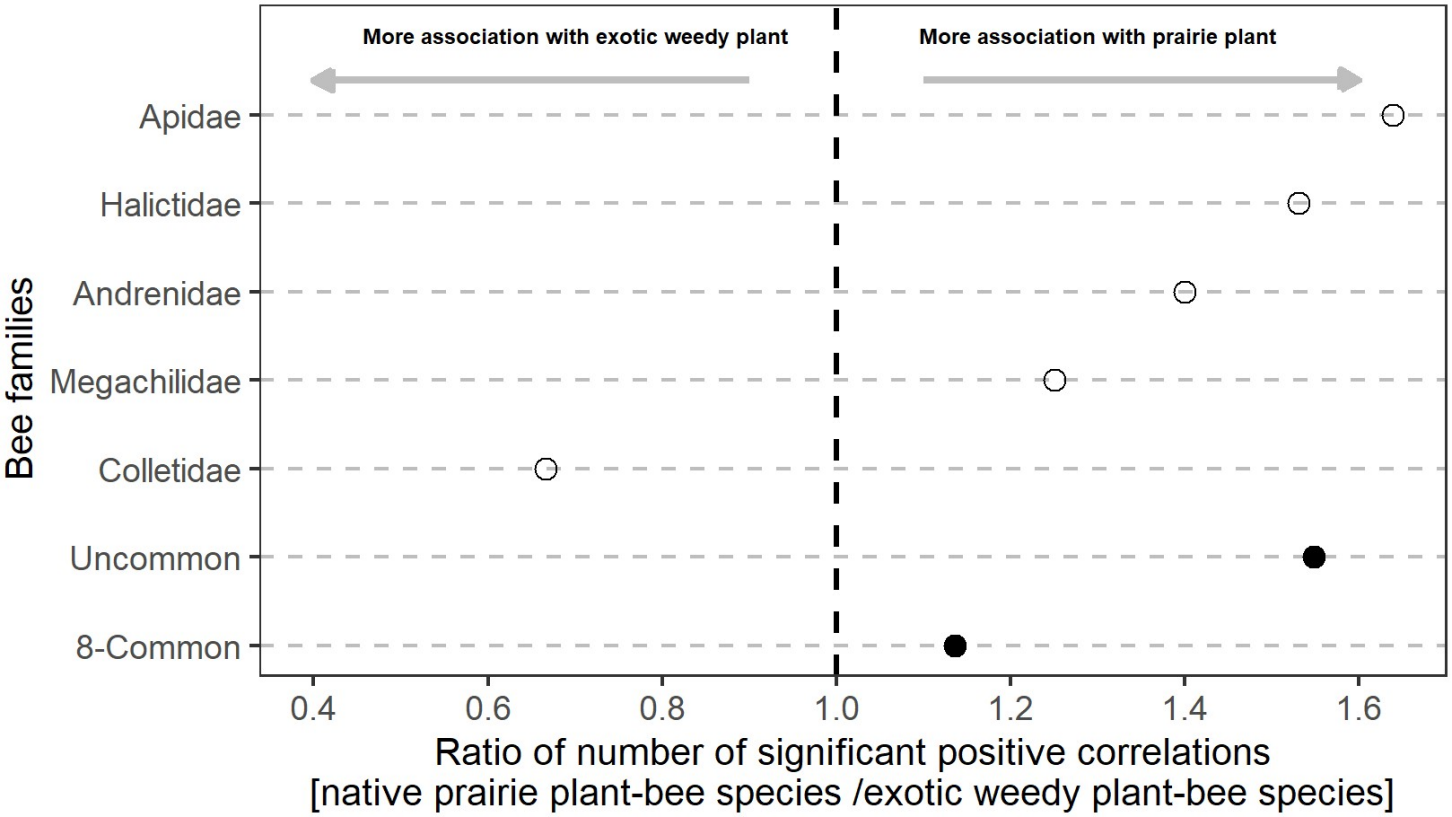
The ratio of the number of significant positive correlations between the native prairie and exotic/weedy species for bee families and bee abundance groups. The dashed line represents the value at which the number of correlations between bees and native prairie and exotic/weedy species are equal. Values>1 and <1 indicate the greater affinity with native prairie and exotic/weedy species, respectively.

The ratio number of significant positive correlations for each group of bees with native prairie forbs to that of exotic/weedy plants indicates the association relationship between various groups of bees and different types of vegetation. As depicted in Fig 4, the ratios > 1 represent a greater correlation between native prairie plant species and a group of bees, whereas the ratios < 1 suggest a weaker correlation with native plant species (or stronger relationship with exotic/weedy species).

Both the common and uncommon bee species were associated more with native prairie forbs than exotic/weedy ones. However, the ratio for common bee species was closer to 1, indicating that common bees were nearly equally correlated with prairie and exotic weedy forbs (Fig 4). Among bee families, the abundant bee families including both the common and uncommon bee species (Apidae and Halictidae) had relatively greater ratios compared to uncommon families (Megachilidae, Colletidae and Andrenidae). The abundant bee families were more closely associated with prairie forb species. Among families of uncommon bee species with the fewest individuals, the Andrenidae (134) and Megachilidae (31) were associated more closely with the native plant species. The Colletidae (35) was the only bee family that was more closely associated with exotic/weedy forb species (Fig 4).

### Plant and bee community correlations

Pair-wise correlations between each forb and bee species provided a means of assessing interactions between bee and plant species. By identifying the significant positive correlations, we were able to demonstrate the proportion of such correlations between each plant and bee family (Table 2). The two most speciose plant families of forb species, the Asteraceae (21 species) and Fabaceae (10 species), also had the highest cover when proportional cover values of all species within that family were accumulated (5.167 and 1.242, respectively) and the highest proportions of significant positive correlations across all bee families, 0.484 and 0.208, respectively. The Amaranthaceae, although represented by only 3 exotic/weedy species, had the next highest proportional cover (1.049), but its proportion of significant positive correlations among all bee families was low (0.024). The Lamiaceae, Apiaceae and Apocynaceae, which were mostly comprised of native forb species, were each represented by few species (1–4) but had notably high correlations with families of uncommon bees. Furthermore, several plant families of few species and proportionately low cover were positively correlated with some, or all, of the bee families composed of uncommon species.

**Table 2:** The proportion of significant positive correlations between species within each plant family and each bee family across all plant and bee communities collected over the course of the experiment (May-August of 2016 and 2017). Plant families among strips are presented in order of proportional cover (proportion of the ground area covered by each family) within 1 m^2^ and bee families with the numbers in the parentheses provide (number of species that were collected); and (relative abundance of species collected from that family within the entire bee community). As the proportions for a crossed bee-plant family increases the support of that plant family for that bee family rises. Rises in the total proportions of a plant family indicates the larger support of plant family for bee community and the increases in the total proportion of a bee family indicates higher support of entire plant community for that bee family.

		Proportion of significant positive correlations between species of each plant and bee family					
Plant family followed by no. species of prairie (P), exotic or weedy native (EW)	Plant family prop. cover	Halictidae (26); (0.848)	Apidae (27); (0.092)	Andrenidae (17); (0.041)	Colletidae (5); (0.011)	Megachillidae (7); (0.008)	Totals
Asteraceae (12P) (9EW)	5.167	0.138	0.141	0.122	0.041	0.043	0.484
Fabaceae (6P) (4EW)	1.242	0.060	0.053	0.045	0.012	0.038	0.208
Amaranthaceae (3EW)	1.049	0	0.019	0	0.005	0	0.024
Polygonaceae (2EW)	0.615	0.002	0.002	0	0	0	0.005
Lamiaceae (1P)	0.491	0.017	0.014	0.010	0	0.002	0.043
Apiaceae (2P) (2EW)	0.488	0.031	0.029	0.029	0.007	0.010	0.105
Plantaginaceae (1EW)	0.201	0.002	0	0	0	0	0.002
Onagraceae (1P)	0.088	0	0.002	0	0	0	0.002
Brassicaceae (2EW)	0.082	0.005	0	0	0	0	0.005
Malvaceae (1EW)	0.066	0	0.007	0	0	0	0.007
Apocynaceae (3P)	0.053	0.010	0.017	0.007	0.000	0.005	0.038
Rosaceae (1P)	0.014	0.002	0	0.005	0.000	0.002	0.010
Oxalidaceae (1EW)	0.013	0.005	0.002	0.005	0.002	0.002	0.017
Urticaceae (1E)	0.013	0.002	0.002	0.005	0.005	0.002	0.017
Scrophulariaceae (1EW)	0.010	0.005	0.005	0.005	0.002	0.002	0.019
Ranunculaceae (2P)	0.007	0.002	0.002	0.005	0.000	0	0.010
Euphorbiaceae (1EW)	0.004	0.000	0.005	0	0	0	0.005
Totals		0.282	0.301	0.237	0.074	0.106	

**Fig 5:**
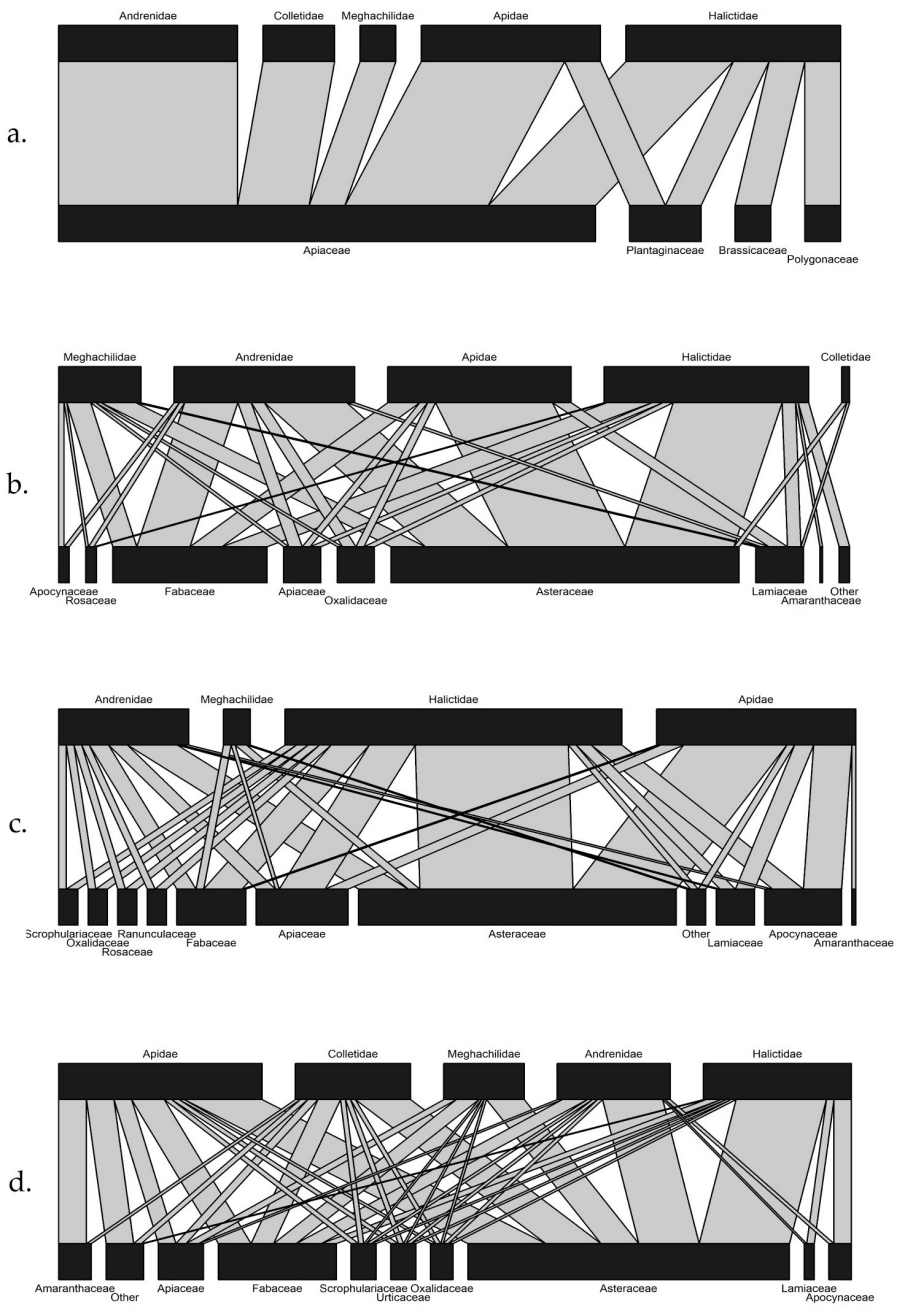
Co-occurrence networks (significant positive correlations) of bee and plant species, within their respective families, among prairie strips by month: (a) May, (b) June, (c) July, and (d) August. The width of the connections from one community to another represents the number of significant positive correlations between bee and plant species (r> 0 and *P*< 0.05).

The co-occurrence networks of bee and plant species for each month during the season can provide insight into the phenology of flowering and activity of bees. In May (Fig 5a), the Apiaceae, represented by a single native prairie species, *Zizia aurea* (L.) W.D.J. Koch (S2 Table), can be seen to have wide lanes of positive correlations with members of all active families including families containing uncommon bee species in the Andrenidae, Colletidae, and Megachilidae (Fig 1). In addition, three exotic species within other plant families, *Polygonum pensylvanicum* L. (Polygonaceae), *Veronica perigrina* L. (Scrophulariaceae), and *Capsella bursa-pastoris* (L.) Medik (Brassicaceae), have high numbers of positive correlations with members of the families that include common bees, the Halictidae and Apidae. By June (Fig 5b), the Apiaceae continued to be positively correlated with species in all bee families. At this time, the Asteraceae, represented by 9 species (6 native and 3 exotic), became widely correlated with all active bee families, followed by the Fabacae (3 exotic and 3 native species), Lamiaceae (1 native species: *Monarda fistulosa* L.), and Oxalidaceae, which was composed entirely of 1 exotic species, *Oxalis stricta* L. The most common bees, members of the Halictidae, showed strong levels of co-occurrence with all the plant families throughout the season as did the members of the other family containing common bees, the Apidae, except in July. At this time, four plant families had wide lanes of correlation with the Andrenidae, a family including only uncommon bees. As the season progressed from May to August the number of plant families positively correlated with bees continuously increased.

## Discussion

This study produced data concerning how strips of reconstructed native prairie vegetation placed in corn and soybean fields effectively sustained bee communities and restored important pollinators. Our results indicated that placing prairie strips in intensively managed agricultural landscapes could compensate for impoverishment of the floral resources in these landscapes that are characterized by domination of crops with no dependency on pollinators.

As we predicted, native prairie strips enhanced bee abundance, species richness and diversity of the entire bee community, with increases in both common and uncommon species. Generally, the level of complexity within the landscape did not affect the positive impact of prairie strips on most groups of bees. But for common bee species richness, less complex landscapes showed larger positive impacts from prairie strips than that of the more complex landscapes. In order to achieve a better understanding of this effect and to evaluate the effectiveness of strip habitat implementation, we compared our results with other studies that assessed the efficiency of habitat enhancement practices within the context of surrounding landscapes [14,24]. This was performed by transforming the Shannon landscape diversity index used in the present study to the proportion of non-crop area (see S5 Table).

The proportion of non-crop area in our study ranged from 8% to 69%, which was comparable with scales in studies of Kleijn et al. and Tscharntke’s et al. [14,24]. Within the ranges of non-crop area in our landscape, benefits that we detected for prairie strips were not always similar to the efficiency of management practice reported by the previous studies [e.g., 14,24]. Unlike previous investigations [15,24], in this study we obtained a surprisingly high benefit from prairie strips for increasing common bee species abundance across all ranges of landscape (more than 8% but less than 69%), that according to Tscharntke’s et al. should be defined as “simple” to “complex” landscapes. Within the lowest range of non-crop area there was an effective enhancement in the richness of common bee species including 8-common and the 9-common, the category in which we included the *Lasioglossum* (Dialictus) sp. More importantly, among these common bee species, four crucial pollinators including *Augochlorella aurata* (Smith); *Halictus ligatus* (Say); *Melissodes bimaculatus* (Lepeletier); *Melissodes trinodis* (Robertson), were restored by implementing prairie strip habitats. Unlike previous studies [15,24], the positive influence of prairie strips in our study was pronounced throughout all ranges of landscape complexities by disproportionate rise in the abundance of common bee species. However, the response of common bee species richness to the prairie strips at the highest level of complexity, i.e., approximately 69% of non-crop area was similar to those of Kleijn et al. and Tscharntke’s et al. Within the most complex landscape, there was no effect from prairie strips on common bee species richness. Nevertheless, we noticed that uncommon bee species consistently benefited from prairie strips in all landscapes and were not impacted by the simplicity or complexity of the landscape, which is in in agreement with other studies [14,15].

The possible enhancement or restoration of common bee species within a less complex landscape is an important factor for evaluating the efficiency and success of a conservation strategy [14]. This is because the rise in common bee species is linked with increased production of crops [6,8,10,14,19,20,22,69,70] that depend on pollinators (e.g., blue-berries, clover, tomatoes), estimated to be worth billions of dollars annually [71,72]. Even though uncommon species may not be as important for crop pollination as common species [3,4,14], conservation practices that support uncommon bee species may augment pollination services to some crops as well as endangered plant species [e.g., 8,10,17–20].

Furthermore, comparing our results in the context of “intermediate landscape-complexity hypothesis” presented by Tscharntke’s et al [24], we determined that prairie strips were highly effective in enhancing the bee communities. Tscharntke et al.’s hypothesis states that the effectiveness of local biodiversity conservation management is lowest either when the landscape diversity (complexity) is extremely low in “cleared landscapes” (<1% non-crop coverage) or very high in “complex landscapes” (>20% non-crop coverage). Management is most effective at an intermediate level of landscape complexity, coined the “simple landscape” (1–20% non-crop coverage), with the possibility of shifting this threshold in different regions of the world. According to our results prairie strips delivered maximum effectiveness within all ranges of the complexities of our landscapes, except for the highest range (approximately 69%). Tscharntke et al. [24] noted that the maximum benefit of conservation management is achieved only in the “simple landscape.” But our study showed that the threshold levels of “simple landscapes” can shift from 1–20% non-crop area to higher levels (i.e., 8–69% non-crop area, Fig 3). This raises the question: why this might have occurred in the landscapes of our study?

Part of the discrepancies between our study and others might be attributed to a lower proportion of flowering crops providing pollen and nectar for bees [1,2] in our study landscapes. Furthermore, the effect size of a conservation practice, e.g. the rise in common and uncommon bee species, might depend on the initial status of the bee community and its baseline characteristics (e.g. richness) at a local scale. Bee communities in our study were notably depauperate and low in species-richness, i.e., 30 genera and subgenera, in comparison with that of the most bee depauperate region in the world, e.g., the Oriental faunal region with 89 genera and sub-genera, and the other regions such as sub-Saharan Africa with175 bee species and even the North America, South America, and Europe [3,14,73]. We believe these factors taken together enhanced the effectiveness of the prairie strip conservation practice in our landscape in comparison with other regions of the world.

As we expected, similar to other studies [e.g., 14,15,74,75], we found that the bee community was enhanced by increasing the abundance of blooming forbs. Unlike previous investigations, we found that both common and uncommon bee species had stronger associations with native forbs than exotic/weedy forb species. Although the strong connections found between uncommon bees and native prairie plants were expected, high associations between common bees and native plants were surprising. The more robust associations of uncommon bees with native prairie species than with exotic plant species could be related to features of prairie strip habitat including provision of pollen, nectar and nesting sites [2,28,15,40]. Common species are often generalist pollinators and are expected to have associations with weedy species [76–78] due to the extended periods of activity from multiple generations within a season. This can be harmonized with spatio-temporal availability of weeds throughout the landscape [77,79]. But the responses we determined in this study indicate that native plant species were more favorable than exotic weedy species for both common and uncommon bees.

The effects we observed in this study were from prairie strips 1–3 years after establishment with nearly equal numbers of native and exotic plant species and similar mean cover/m^2^. We expect that as the plant communities of the strips mature over time, the abundance of native forbs increases [80]. Consequently, the dependence of bees on exotic plants will likely shift to native prairie species. The alteration of visitation toward native plant species has been reported by Morandin and Kremen [22], who found that as time progressed maturing hedgerows become dominated by native plants and the majority of bee species changed their preference of visitation from exotic to native plant species. Thus, over time, prairie strips may support a broader range of bee species than what we observed, particularly early in the season, as was found by Moorhouse [81].

Using the seasonal correlation-based co-occurrence networks explored in our study, we were able to identify individual plant species as possible keystones supporting bee species throughout the season. Three plant families, the Apiaceae, Asteraceae and Fabaceae, were especially important for increasing the abundant bee families. Our findings suggest that the native species *Zizia aurea (L*.*)* W.D.J. Koch, may be an important floral resource early in the season for the entire bee community. This supports the findings of Harmon-Threatt and Hendrix, who also concluded that *Z*. *aurea* as one of four key species supporting prairie bees [40], especially beginning in the season. Among the late-season-blooming Asteraceae, two native prairie species included in our study, *Ratibida pinnata* (Vent.) Barnhart and *Solidago rigida* L., and two exotics, *Cirsium arvense* (L.) Scop., and *Taraxacum officinale* (L.) Weber ex F.H. Wigg, were highly correlated with bee species. These four species were the most abundant species within the prairie vegetation, and possibly the two native plant species could be as attractive as exotic weedy species. Furthermore, our study suggests the native plant of *R*. *pinnata*, or its regional equivalent, is a useful species to be included in plant communities intended to support bees [40].

Co-occurrence networks explored in this study can possibly be a complementary tool for observed bee-plant networks, since this method might resolve the biases accompanied by networks of visitation [e.g, 42]. We acknowledge, however, that co-occurrence networks should be used with caution so as to not over-estimate some bee-plant interactions with no biological meaning. We suggest using co-occurrence networks along with visitation networks whenever possible to achieve a more accurate and comprehensive picture of the structural complexity of bee-plant interactions.

Here we showed that prairie strips occupying 10–12% of the area of crop fields constituted an efficacious conservation strategy for restoring and sustaining bee communities, including both uncommon and common bee species, within the simplified landscapes of Midwest US. This effect was found even within more complex landscapes with >20% of non-crop area, where we did not expect to gain benefits from the conservation practice. The latter result may be linked to the domination of landscape with crops that have no dependency on insect pollinators in addition to the depauperate status of bee communities within the studied area. We conclude that strips of native prairie habitat and the forb species they contain can compensate for the scarcity of floral resources and buffer the decline of bees in agriculturally dominated landscapes in the Midwest U.S.

## Supporting information

S1 Table List of bee species with family, abundance (U = uncommon, C = Common), the month(s) of activity and number of individuals collected (No.) over the entire study (2016–2017). (DOCX)

S2 Table Forb species of prairie strip, their family, and mean cover over the entire study (2016–2017), designation as native (N), exotic or weedy (EW), and seed origin (M-seed mix, B-seed bank). (DOCX)

S3 Table Shannon landscape diversity, H’s = -∑((pi) × ln (pi)), index averages within a 3-km radius of each treatment at each site over the entire study along with differences between treatments, associated *t*_df_ (*t*-test) and *p* values and the overall *t*_df_ and *p* value of differences. (DOCX)

S4 Table *F*_df_ statistics and *p* values and of bee abundance and species richness of each family during May-August of 2016 and 2017 from all trapping methods. (DOCX)

S5 Table Categories of vegetation cover used for calculation of Shannon landscape diversity within 3-km radius of landscape surrounding each treatment at each site. Non-crop classes are marked with X.
(DOCX)

S6 Table *F*_df (regression)_ statistics and *p* value of the regression analysis (ANOVA) for different classes of bees (All bees, 8-common, 9-common, uncommon) separately by abundance and species richness. (DOCX)
