# Supplementary material for: Strips of prairie vegetation placed within row crops can sustain native bee communities
Source: PLoS One. 2020 Oct 29;15(10):e0240354. doi: 10.1371/journal.pone.0240354 (PMC7595394; doi:10.1371/journal.pone.0240354)
Supplement: S1 Table — (DOCX) [file pone.0240354.s001.docx]

**S1 Table.** List of bee species with family, abundance (U=uncommon, C=Common), the month(s) of activity and number of individuals collected (No.) over the entire study (2016-2017).

| **Species** | **Family** | **Abundance** | **Active** | **No.** |
| --- | --- | --- | --- | --- |
| *Agapostemon sericeus* (Forster, 1771) | Halictidae | U | May - August | 33 |
| *Agapostemon angelicus* Cockerell, 1924 | Halictidae | U | June | 4 |
| *Agapostemon splendens* (Lepeletier, 1841) | Halictidae | U | July | 4 |
| *Agapostemon texanus* Cresson, 1872 | Halictidae | U | May - August | 40 |
| *Agapostemon virescens* (Fabricius, 1775) | Halictidae | C | May - August | 231 |
| *Andrena accepta* Viereck, 1916 | Andrenidae | U | August | 1 |
| *Andrena alleghaniensis* Viereck, 1907 | Andrenidae | U | May | 1 |
| *Andrena commoda* Smith, 1879 | Andrenidae | U | May - June | 27 |
| *Andrena cressonii cressonii* Robertson, 1891 | Andrenidae | U | June | 14 |
| *Andrena erigeniae* Robertson, 1891 | Andrenidae | U | May | 11 |
| *Andrena helianthiformis* Viereck & Cockerell, 1914 | Andrenidae | U | June - July | 4 |
| *Andrena rudbeckiae* Robertson, 1891 | Andrenidae | U | June - July | 22 |
| *Andrena sayi* Robertson, 1891 | Andrenidae | U | May - June | 6 |
| *Andrena* sp. | Andrenidae | U | May - June | 11 |
| *Andrena wilkella* (Kirby, 1802) | Andrenidae | U | June - July | 2 |
| *Andrena zizae* Robertson, 1891 | Andrenidae | U | May | 1 |
| *Anthophora terminalis* Cresson, 1869 | Apidae | U | July | 1 |
| *Anthophora walshii* Cresson, 1869 | Apidae | U | July | 1 |
| *Apis mellifera* Linnaeus, 1758 | Apidae | U | May - August | 28 |
| *Ashmeadiella bucconis*  (Say, 1837) | Megachilidae | U | May, July, August | 7 |
| *Ashmeadiella* sp. | Megachilidae | U | June | 1 |
| *Augochlora pura* (Say, 1837) | Halictidae | U | July, August | 5 |
| *Augochlorella aurata* (Smith, 1853) | Halictidae | C | May - August | 298 |
| *Augochlorella persimilis* (Viereck, 1910) | Halictidae | U | May, August | 7 |
| *Augochlorella* sp. | Halictidae | U | May - August | 23 |
| *Augochloropsis metallica* (Fabricius, 1793) | Halictidae | U | May, June | 6 |
| *Augochloropsis sumptuosa* (Smith, 1853) | Halictidae | U | June | 1 |
| *Bombus bimaculatus* Cresson, 1863 | Apidae | U | May, July | 4 |
| *Bombus griseocollis* (De Geer, 1773) | Apidae | U | July, August | 3 |
| *Bombus impatiens* Cresson, 1863 | Apidae | U | May - August | 12 |
| *Bombus pensylvanicus* (De Geer, 1773) | Apidae | U | May - August | 22 |
| *Bombus auricomus* (Robertson, 1903) | Apidae | U | May | 1 |
| *Bombus vagans* Smith, 1854 | Apidae | U | June, July | 2 |
| *Calliopsis andreniformis* Smith, 1853 | Andrenidae | U | June - August | 14 |
| *Ceratina calcarata* Robertson, 1900 | Apidae | U | May, June | 14 |
| *Ceratina dupla* Say, 1837 | Apidae | U | May, June | 5 |
| *Ceratina mikmaqi* Rehan and Sheffield, 2011 | Apidae | U | May | 1 |
| *Ceratina strenua* Smith, 1879 | Apidae | U | May, June | 3 |
| *Coelioxys octodentata* Say, 1824 | Megachilidae | U | August | 1 |
| *Colletes inaequalis* Say, 1837 | Colletidae | U | July | 1 |
| *Colletes simulans armatus* Patton, 1879 | Colletidae | U | August | 1 |
| *Eucera dubitata* (Cresson, 1878) | Apidae | U | May | 1 |
| *Eucera hamata* (Bradley, 1942) | Apidae | C | May, June, July | 140 |
| *Eucera rosae* (Robertson, 1900) | Apidae | U | May | 1 |
| *Halictus confusus* Smith, 1853 | Halictidae | C | May - August | 119 |
| *Halictus ligatus* Say, 1837 | Halictidae | C | May - August | 370 |
| *Halictus parallelus* Say, 1837 | Halictidae | C | May - August | 67 |
| *Halictus rubicundus* (Christ, 1791) | Halictidae | U | May - August | 55 |
| *Halictus* sp. | Halictidae | U | June | 2 |
| *Halictus tripartitus* Cockerell, 1895 | Halictidae | U | May - August | 27 |
| *Hoplitis pilosifrons* (Cresson, 1864) | Megachilidae | U | June | 1 |
| *Hylaeus affinis*  (Smith, 1853) | Colletidae | U | May, June, August | 13 |
| *Hylaeus mesillae* (Cockerell, 1896) | Colletidae | U | May, August | 20 |
| *Hylaeus sparsus* (Cresson, 1869) | Colletidae | U | August | 1 |
| *Lasioglossum* (*Dialictus*) sp. | Halictidae | - | May - August | 2969 |
| *Lasioglossum* (Evylaceus, s.l.) | Halictidae | U | June | 1 |
| *Lasioglossum* s.s. *acuminatum* McGinley, 1986 | Halictidae | U | May | 5 |
| *Lasioglossum* s.s. *athabascense* (Sandhouse, 1933) | Halictidae | U | June, July | 2 |
| *Lasioglossum* s.s. *coriaceum* (Smith, 1853) | Halictidae | U | May, June, August | 34 |
| *Lasioglossum* s.s. *fuscipenne* (Smith, 1853) | Halictidae | U | May, June, July | 6 |
| *Lasioglossum* s.s. *paraforbesii* McGinley, 1986 | Halictidae | U | May, June | 4 |
| *Lasioglossum* s.s. *zonulum* (Smith, 1848) | Halictidae | U | August | 2 |
| *Lithurgopsis littoralis* (Cockerell, 1917) | Megachilidae | U | June | 1 |
| *Megachile brevis* Say, 1837 | Megachilidae | U | June - August | 18 |
| *Megachile latimanus* Say, 1823 | Megachilidae | U | June, August | 2 |
| *Melissodes bidentis* Cockerell, 1914 | Apidae | U | June - August | 6 |
| *Melissodes bimaculatus* (Lepeletier, 1825) | Apidae | C | June - August | 164 |
| *Melissodes communis* Cresson, 1878 | Apidae | U | June - August | 23 |
| *Melissodes comptoides* Robertson, 1898 | Apidae | U | July | 2 |
| *Melissodes coreopsis* Robertson, 1905 | Apidae | U | August | 1 |
| *Melissodes desponsus* Smith, 1854 | Apidae | U | June, August | 11 |
| *Melissodes druriella* (Kirby, 1802) | Apidae | U | June - August | 7 |
| *Melissodes menuachus* Cresson, 1868 | Apidae | U | August | 1 |
| *Melissodes* sp. | Apidae | U | August | 3 |
| *Melissodes trinodis* Robertson, 1901 | Apidae | C | June - August | 100 |
| *Nomada* sp. | Apidae | U | May - July | 3 |
| *Nomia howardi* Crawford, 1911 | Halictidae | U | June | 1 |
| *Nomia nortoni* Cresson, 1868 | Halictidae | U | July | 1 |
| *Nomia universitatis* Cockerell, 1908 | Halictidae | U | June | 1 |
| *Protandrena bancrofti* Dunning, 1897 | Andrenidae | U | June | 1 |
| *Pseudopanurgus albitarsis* (Cresson, 1872) | Andrenidae | U | June | 2 |
| *Pseudopanurgus compositarum* (Robertson, 1893) | Andrenidae | U | August | 1 |
| *Pseudopanurgus helianthi* Mitchell, 1960 | Andrenidae | U | June, August | 14 |
| *Pseudopanurgus labrosiformis* (Robertson, 1898) | Andrenidae | U | August | 1 |
| *Pseudopanurgus* sp. | Andrenidae | U | June | 1 |
| *Sphecodes arctoSphecodes ms name* (a.k.a. dichros group) | Halictidae | U | July | 2 |
| *Sphecodes* sp. | Halictidae | U | June, August | 4 |
| *Triepeolus lunatus* (Say, 1824) | Apidae | U | July | 1 |
| *Xylocopa virginica* (Linnaeus, 1771) | Apidae | U | May - June | 2 |
