# Supplementary material for: Strips of prairie vegetation placed within row crops can sustain native bee communities
Source: PLoS One. 2020 Oct 29;15(10):e0240354. doi: 10.1371/journal.pone.0240354 (PMC7595394; doi:10.1371/journal.pone.0240354)
Supplement: S2 Table — (DOCX) [file pone.0240354.s002.docx]

**S2 Table**. Prairie strip plant species, family, mean cover over the entire study (2016-2017), designation as native (N), exotic or weedy (EW), and seed origin (M-seed mix, B-seed bank).

| Species | Family | Cover/m^2^ | Native or  Exotic/weedy | Origin | Bloom period |
| --- | --- | --- | --- | --- | --- |
| *Rudbeckia hirta* L. | Asteraceae | 1.445 | N | M | June - August |
| *Ratibida pinnata* (Vent.) Barnh. | Asteraceae | 1.072 | N | M | June - August |
| *Trifolium repens* L. | Fabaceae | 0.832 | EW | B | May - August |
| *Chenopodium album* L. | Amaranthaceae | 0.701 | EW | B | July - August |
| *Conyza canadensis* (L.) Cronq. | Asteraceae | 0.656 | EW | B | June - August |
| *Polygonum pensylvanicum* L. | Polygonaceae | 0.589 | EW | B | May - August |
| *Cirsium arvense* (L.) Scop. | Asteraceae | 0.516 | EW | B | June -July |
| *Heliopsis helianthoides* (L.) Sweet | Asteraceae | 0.509 | N | M | June - August |
| *Monarda fistulosa* L. | Lamiaceae | 0.491 | N | M | June - August |
| *Taraxacum officinale* (L.) ex. F.H.Wigg | Asteraceae | 0.378 | EW | B | May - August |
| *Amaranthus rudis* J. D. Sauer | Amaranthaceae | 0.343 | EW | B | June, August |
| *Zizia aurea (L.) W.D.J. Koch* | Apiaceae | 0.222 | N | M | May - July |
| *Daucus carota* (L.) | Apiaceae | 0.221 | EW | B | May - August |
| *Trifolium pratense* L. | Fabaceae | 0.218 | EW | B | May - August |
| *Veronica peregrina* L. | Plantaginaceae | 0.201 | EW | B | May - August |
| *Symphyotrichum ericoides (L.) G.L. Nesom* | Astreceae | 0.139 | N | M | June - August |
| *Chamaecrista fasciculata* (Michx.) Greene | Fabaceae | 0.139 | N | M | July - August |
| *Echinacea pallida* (Nutt.) Nutt. | Asteraceae | 0.108 | N | M | June - August |
| *Lactuca serriola* L. | Asteraceae | 0.089 | EW | B | July - August |
| *Oenothera biennis* L. | Onagraceae | 0.088 | N | M | July - August |
| *Cirsium arvense f albiflorum (E.L.Rand & Redfield) R.Hoffman.* | Asteraceae | 0.072 | EW | B | June - August |
| *Abutilon theophrasti* Medik. | Malvaceae | 0.066 | EW | B | July - August |
| *Solidago rigida* L. | Asteraceae | 0.060 | N | M | July - August |
| *Capsella bursa-pastoris* (L.) Medik. | Brassicaceae | 0.055 | EW | B | May - June |
| *Asclepias tuberosa* L. | Apocynaceae | 0.043 | N | M | June - August |
| *Eryngium yuccifolium* Michx. | Apiaceae | 0.043 | N | M | July - August |
| *Cirsium vulgare* (Savi) Ten. | Asteraceae | 0.031 | EW | B | July - August |
| *Thlaspi arvense* L. | Brassicaceae | 0.027 | EW | B | June |
| *Symphyotrichum Novaenovae-angliae (L.) G.L. Nesom* | Asteraceae | 0.027 | N | M | August |
| *Rumex crispus* L. | Polygonaceae | 0.026 | EW | B | May |
| *Achillea millefolium L.* | Asteraceae | 0.026 | EW | B | July - August |
| *Desmodium canadense* (L.) DC. | Fabaceae | 0.014 | N | M | June |
| *Solidago speciosa* Nutt. | Asteraceae | 0.014 | N | M | August |
| *Drymocallis arguta* (Pursh) Rydb. | Rosaceae | 0.014 | N | M | June - July |
| *Urtica dioica* L. | Urticaceae | 0.013 | EW | B | August |
| *Oxalis stricta* L. | Oxalidaceae | 0.013 | EW | B | May - August |
| *Medicago sativa* L. | Fabaceae | 0.011 | N | B | August |
| *Dalea purpurea* Vent. | Fabaceae | 0.011 | N | M | June - July |
| *Verbascum thapsus* L. | Scrophulariaceae | 0.010 | EW | B | July - August |
| *Lotus corniculatus* L. | Fabaceae | 0.009 | EW | B | August |
| *Artemisia ludoviciana* Nutt. | Asteraceae | 0.008 | N | M | July - August |
| *Asclepias syriaca* L. | Apocynaceae | 0.007 | N | M | July |
| *Amaranthus retroflexus* L. | Amaranthaceae | 0.005 | EW | B | August |
| *Leucanthemum vulgare* Lam. | Astercaeae | 0.005 | N | M | August |
| *Baptisia alba* (L.) Vent. | Fabaceae | 0.004 | N | M | August |
| *Solidago canadensis* L. | Asteraceae | 0.004 | N | M | July - August |
| *Ambrosia artemisiifolia* L. | Asteraceae | 0.004 | EW | B | August |
| *Euphorbia maculata* L. | Euphorbiaceae | 0.004 | EW | B | August |
| *Aquilegia canadensis* L. | Ranunculaceae | 0.004 | N | M | July |
| *Melilotus officinalis* (L.) Pall. | Fabaceae | 0.003 | EW | B | June - July |
| *Delphinium viridescens* Lieberg | Ranunculaceae | 0.003 | N | M | July |
| *Asclepias incarnata* L. | Apocynaceae | 0.003 | N | M | June |
| *Xanthium strumarium* L. | Asteraceae | 0.002 | EW | B | August |
| *Pastinaca sativa* L. | Apiaceae | 0.002 | EW | B | July |
| *Silphium laciniatum L.* | Asteraceae | 0.002 | N | M | July - August |
| *Desmanthus illinoensis* (Michx.) MacMillan | Fabaceae | <0.000 | N | M | August |
