# Supplementary material for: Strips of prairie vegetation placed within row crops can sustain native bee communities
Source: PLoS One. 2020 Oct 29;15(10):e0240354. doi: 10.1371/journal.pone.0240354 (PMC7595394; doi:10.1371/journal.pone.0240354)
Supplement: S3 Table — (DOCX) [file pone.0240354.s003.docx]

**S3 Table.** Shannon landscape diversity, H’s =-∑((pi) × ln (pi)), index averages within a 3-km radius of each treatment at each site over the entire study along with differences between treatments, associated *t*_df_ (*t*-test) and *p* values and the overall *t*_df_  and *p* value of differences.

| Shannon landscape diversity | Guthrie | | Linn | | Marshall | | Pottawattamie | |
| --- | --- | --- | --- | --- | --- | --- | --- | --- |
|  | Strip | Control | Strip | Control | Strip | Control | Strip | Control |
| Average over two years | 1.586 | 1.586 | 1.005 | 1.153 | 1.471 | 1.486 | 1.254 | 1.297 |
| Differences between treatments | 0 | | 0.148 | | 0.015 | | 0.043 | |
| Individual site | *t*_1_= 0.007 | *p* = 0.999 | *t*_1_ *=* 25.86 | *p* = 0.021 | *t*_1_*=* 0.654 | *p* = 0.764 | *t*_1_*=* 7.763 | *p* = 0.129 |
| Overall | *t*_7_ = 2.28 | | | | *p* = 0.06 | | | |
