# Supplementary material for: Strips of prairie vegetation placed within row crops can sustain native bee communities
Source: PLoS One. 2020 Oct 29;15(10):e0240354. doi: 10.1371/journal.pone.0240354 (PMC7595394; doi:10.1371/journal.pone.0240354)
Supplement: S4 Table — (DOCX) [file pone.0240354.s004.docx]

**S4 Table.** *F*_df_ statistics and *p* values and of bee abundance and species richness of each family during May-August of 2016 and 2017 from all trapping methods.

|  | **Abundance** | | **Species richness** | |
| --- | --- | --- | --- | --- |
|  |  | |  | |
| **Family** | ***p* value** | ***F*_3,36_** | ***p* value** | ***F*_3,36_** |
| Apidae | 0.006 | 4.90 | 0.008 | 4.51 |
| Halictidae | 0.028 | 3.41 | 0.561 | 0.70 |
| Andrenidae | 0.025 | 3.51 | 0.037 | 3.13 |
| Colletidae | 0.092 | 2.31 | 0.145 | 1.91 |
| Megachilidae | 0.204 | 1.61 | 0.335 | 1.17 |
