# Supplementary material for: Strips of prairie vegetation placed within row crops can sustain native bee communities
Source: PLoS One. 2020 Oct 29;15(10):e0240354. doi: 10.1371/journal.pone.0240354 (PMC7595394; doi:10.1371/journal.pone.0240354)
Supplement: S5 Table — Non-crop classes are marked with X. (DOCX) [file pone.0240354.s005.docx]

**S5 Table.** Categories of vegetation cover used for calculation of Shannon landscape diversity within 3-km radius of landscape surrounding each treatment at each site. Non-crop classes are marked with X.

| Categories of landscape vegetation | Non-crop category |
| --- | --- |
| Alfalfa |  |
| Clover/Wildflowers |  |
| Corn |  |
| Deciduous Forest | X |
| Evergreen Forest | X |
| Fallow/Idle Cropland | X |
| Grass/Pasture | X |
| Herbaceous Wetlands | X |
| Oats |  |
| Other Hay/Non Alfalfa |  |
| Pop or Orn Corn |  |
| Rye |  |
| Shrubland | X |
| Soybeans |  |
| Winter Wheat |  |
| Woody Wetlands | X |
