# Supplementary material for: Strips of prairie vegetation placed within row crops can sustain native bee communities
Source: PLoS One. 2020 Oct 29;15(10):e0240354. doi: 10.1371/journal.pone.0240354 (PMC7595394; doi:10.1371/journal.pone.0240354)
Supplement: S6 Table — (DOCX) [file pone.0240354.s006.docx]

**S6 Table.** *F*_df (regression)_ statistics and *p* value of the regression analysis (ANOVA) for different classes of bees (All bees, 8-common, 9-common, uncommon) separately by abundance and species richness.

|  |  | Index | | | |
| --- | --- | --- | --- | --- | --- |
|  |  | Abundance | | Species richness | |
| Class of bees |  | *F*_3,60_ | *p* value | *F*_3,60_ | *p* value |
|  | ANOVA |  |  |  |  |
| All bees | Model | 9.73 | < 0.0001 | 10.93 | < 0.0001 |
|  | Landscape diversity |  | < 0.0001 |  | 0.0017 |
|  | Treatment |  | 0.0006 |  | < 0.0001 |
|  | Landscape diversity x Treatment |  | 0.157 |  | 0.0873 |
|  |  |  |  |  |  |
| 8-common bees | Model | 7.25 | < 0.0003 | 11.89 | < 0.0001 |
|  | Landscape diversity |  | 0.0005 |  | < 0.0001 |
|  | Treatment |  | 0.0014 |  | 0.0003 |
|  | Landscape diversity x Treatment |  | 0.198 |  | 0.0373 |
|  |  |  |  |  |  |
| 9-common bees | Model | 9.18 | < 0.0001 | 11.79 | < 0.0001 |
|  | Landscape diversity |  | < 0.0001 |  | <0.0001 |
|  | Treatment |  | 0.0022 |  | 0.0004 |
|  | Landscape diversity x Treatment |  | 0.1086 |  | 0.0234 |
|  |  |  |  |  |  |
| uncommon bees | Model | 5.44 | 0.0022 | 7.09 | 0.0004 |
|  | Landscape diversity |  | 0.278 |  | 0.265 |
|  | Treatment |  | 0.0002 |  | <0.0001 |
|  | Landscape diversity x Treatment |  | 0.731 |  | 0.812 |
